# Fabrication and Mechanical Behavior of Ex Situ Mg-Based Bulk Metallic Glass Matrix Composite Reinforced with Electroless Cu-Coated SiC Particles

**DOI:** 10.3390/ma10121371

**Published:** 2017-11-30

**Authors:** Xin Wang, Lichen Zhao, Ximei Hu, Yongjian Cheng, Shuiqing Liu, Peng Chen, Chunxiang Cui

**Affiliations:** Key Laboratory for New Type of Functional Materials in Hebei Province, School of Material Science and Engineering, Hebei University of Technology, Tianjin 300130, China; ahaxin@hebut.edu.cn (X.W.); zhlch@hebut.edu.cn (L.Z.); huximei@hebut.edu.cn (X.H.); chengyongjianrest@163.com (Y.C.); liushuiqing0824@126.com (S.L.); m15900225021@163.com (P.C.)

**Keywords:** bulk metallic glass matrix composite, bulk metallic glass, silicon carbide, electroless plating, mechanical properties

## Abstract

Magnesium-based bulk metallic glass matrix composites (BMGMCs) have better plasticity than the corresponding bulk metallic glasses (BMGs); however, their strength and density are often compromised due to the fact that the effective reinforcement phase is mostly plastic heavy metal. For lightweight SiC-particle reinforced BMGMCs, interface wettability and the sharpness of the particles often reduce the strengthening effect. In this work, SiC particles were coated with a thin Cu coating by electroless plating, and added to Mg_54_Cu_26.5_Ag_8.5_Gd_11_ melt in an amount of 5 wt % to prepare a BMGMC. The microstructure of the interface, mechanical behavior and fracture morphology of the BMGMC were studied by scanning electron microscopy and quasi-static compression testing. The results showed that the Cu coating improved the wettability between SiC and the matrix alloy without obvious interfacial reactions, leading to the dispersion of SiC particles in the matrix. The addition of Cu-coated SiC particles improved the plastic deformation ability of Mg_54_Cu_26.5_Ag_8.5_Gd_11_ BMG, proving that electroless plating was an effective method for controlling the interface microstructure and mechanical behavior of BMGMCs.

## 1. Introduction

Compared to traditional magnesium alloys, magnesium-based bulk metallic glasses (BMGs) have higher fracture strength, high elasticity limits and good biodegradability; regarded as having potential applications in many fields [1,2]. In general, BMG has a small characteristic process-zone, which is refers to the plastic process zone at the tip of a crack, and shows localized plastic deformation, as well as exhibiting brittle mechanical behavior [3,4]. The characteristic size of the plastic process zone for Mg-based BMGs is the smallest among BMGs, and seriously limits its further application as a structural material. Making bulk metallic glass matrix composites (BMGMCs) has been proven to be an effective strategy for improving the plasticity of BMGs at room temperature, because the plastic crystalline second phase is able to absorb deformation energy and induce multiple shear banding within the BMG [5,6]. Specifically, the plasticity of the second phase itself is better, and the plastic deformation ability of the composite is further increased. In addition, as the spacing distance and the size of the plastic crystalline second phase are matched to the characteristic size of plastic process zone of the MG matrix, multiple shear banding can be achieved, leading to the global plasticity of the composite [7]. At present, the most effective secondary phase in BMGMCs mostly comprises plastic metals such as W, Mo, Fe, Nb, Ta, etc. [8,9,10,11,12]. However, these additives are generally heavy phases, and will therefore increase the density of the composite, which is not conducive to the lightweight development of composite materials. Furthermore, the introduction of soft or plastic phases will decrease the yield strength [7] and reduce the competitiveness of BMGs with respect to other high-strength materials such as steels [13], titanium alloys [14,15,16] and high entropy alloys [17,18].

For lightweight reinforcements, typical ceramic SiC particles, which are widely used in conventional metal matrix composites, have also been introduced in BMGMCs [19,20,21,22,23,24]. Unfortunately, it is a major challenge to use SiC as an effective reinforcement. SiC particles are more likely to play a role as a source of cracking, due to the large stress concentration caused by their typically multi-angular shape [19]. As a result, SiC particle-reinforced BMGMCs are difficult to achieve effective brittle-to-tough transition. In addition, as a heterogeneous phase in the glass-forming melt, SiC particles will have some chemical and physical interaction with the liquid, when added and stirred into the melt. Such interactions will, in turn, cause some unique metallurgical phenomena, such as wetting and interfacial reactions, which are essential for the preparation of composite materials. In order to improve the wettability and improve the uniformity of the dispersion of SiC particles in the matrix, many expensive devices that require high energy consumption have been used, such as vacuum hot sintering [22] and spark plasma sintering [23,24]; processes that are obviously not conducive to reducing the cost of material preparation. The development of inexpensive methods or simple technologies for the fabrication of SiC composite materials become a new challenge in the field of BMGMCs.

In the present work, aimed at modifying the morphology and shape of SiC particles and improving the wettability between SiC and MG matrix, we used the typical electroless plating method to coat SiC particles with copper. The effects of electroless copper plating on the preparation, microstructure and mechanical deformation behavior of composites based on a typical Mg-based BMG Mg_54_Cu_26.5_Ag_8.5_Gd_11_ [25,26] alloy were studied.

## 2. Materials and Methods

The electroless plating of SiC particles is performed as follows: cleaning (degreasing)→coarsening→sensitizing-activating→plating→filtration→drying. Commercial SiC particles with a purity of 99.9 wt % and a size of 800 mesh (10~20 µm) were sonicated with acetone for 20 min and then washed with deionized water to remove contaminants from the surface. Next, the cleaned particles were immersed in the roasted solution (HF 20 mL/L and NaF 4 g/L) for 15 min to prefabricate a rough surface around the particle. Subsequently, the coarsened SiC particles were put in a sensitizing-activating solution (SnCl_2_∙2H_2_O 30 g/L, HCl 60 mL/L, PdCl_2_ 0.1 g/L and NaCl 160 g/L), stirring for 30 min. Finally, the activated SiC particles were placed in a bath (CuSO_4_∙5H_2_O 25 g/L, HCHO 44 mL/L, C_10_H_14_N_2_Na_2_O_8_∙2H_2_O 24 g/L, KNaC_4_H_4_O_6_∙4H_2_O 16 g/L, K_4_[Fe(CN)_6_] mg/L and C_10_H_8_N_2_ 20 mg/L), plating for about 120 min at a temperature of 40 °C. The pH value of the bath was adjusted to 12.0 with saturated NaOH solution by using a temperature compensation type pH meter (PHS-3CB; Shanghai Yueping Scientific Instrument Co., Ltd., Shanghai, China).

High-purity raw elements Mg (99.99 wt %), Cu (99.99 wt %), Ag (99.999 wt %) and Gd (99.5 wt %) were melted by vacuum induction melting in a high purity graphite crucible to prepare the base alloy with a composition of Mg_54_Cu_26.5_Ag_8.5_Gd_11_ [25,26]. The base alloy ingots were re-melted by induction melting at temperatures ranging 600 °C–800 °C, which was monitored and controlled by a double color laser infrared thermometer. Subsequently, the Cu-coated SiC particles (Wrapped in aluminum foil) were added in the liquid with an addition amount of 5 wt % to fabricate the master alloy SiC/Mg_54_Cu_26.5_Ag_8.5_Gd_11_ ingot under mechanically stirring. The master alloy was then re-melted by introduction melting, and cast into a copper mold to prepare a rod with a diameter of 3 mm and a length of 70 mm (so-called injection casting, the schematic diagram of the device has been described in detail elsewhere [27,28]).

Quasi-static compression tests were performed on an electronic testing (WDW-10KN, Jilin Guanteng Automation Technology Co., Ltd, Changchun, China) machine at a strain rate of 4 × 10^−4^ s^−1^ at room temperature. The compression sample with a diameter of 3 mm and a length of 6 mm was mechanically cut from the as-cast BMGMC rods by a slow diamond cutting machine (SYJ-160, Shenyang Kejing Auto-instrument Co., Ltd, Shenyang, China). For each material, the compression test was performed at least 3 times to verify the reproducibility of experimental results. The fracture surfaces of the fracture samples were analyzed by scanning electron microscope (SEM, Hitachi-S4800; Hitachi, Tokyo, Japan).

In addition, the amorphous structure of the as-cast samples was confirmed by X-ray diffractometry (XRD, Bruker D8 Advance, Cu Kα; Bruker, Billerica, MA, USA). The test samples were cut from the middle part of the as-cast rod. The microstructure of the BMGMC was studied by SEM (Hitachi S-4800, Hitachi, Tokyo, Japan) with an energy dispersive spectrometer (EDS, EDAX, Mahwah, NJ, USA).

## 3. Results

### 3.1. Electroless Cu Plating on SiC Particles

**Figure 1:**
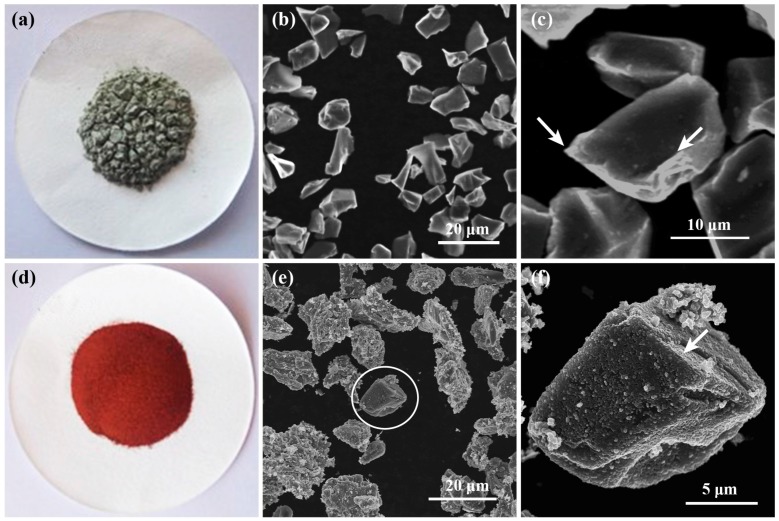
Macroscopic and microscopic morphology of SiC particles of (**a**–**c**) as-received SiC particles and (**d**–**f**) Electroless Cu-coated SiC particles.

Figure 1a displays the optical image of the as-received SiC powder, in which the powders typically show a grey or green color. From the SEM images, as shown in Figure 1b,c, the size of the SiC particles can be seen to have an average value of ~20 µm. The as-received SiC particles have an irregular shape with very sharp corners and edges as marked by the white arrows in Figure 1c. After electroless copper plating, the color of the SiC particles changes to the bronze of typical copper metal, and the fluidity of the powder becomes better, as shown in Figure 1d. Electroless plating of SiC particles is generally non-uniform, due to the tendency of powders to agglomerate in the bath, resulting in different coating thicknesses on different particles. For example, the coating of the SiC particle marked by a white circle in Figure 1e is very thin, and barely visible at low magnification. However, in the high-magnification image—Figure 1f—the SiC particle is completely covered with Cu particles. This indicates that the Cu plating is effective even at the edge of the SiC particles (indicated by the arrow), causing the sharpness of the SiC particles to be reduced despite the relatively thin coating. In addition, the size of the coated SiC particles is not obviously changed, which suggests that the thickness of the coating is very small. In fact, the thickness of the coating can be roughly estimated to be in a range of ~800 nm, according to the magnitude of the mass increase of the coated powder.

**Figure 2:**
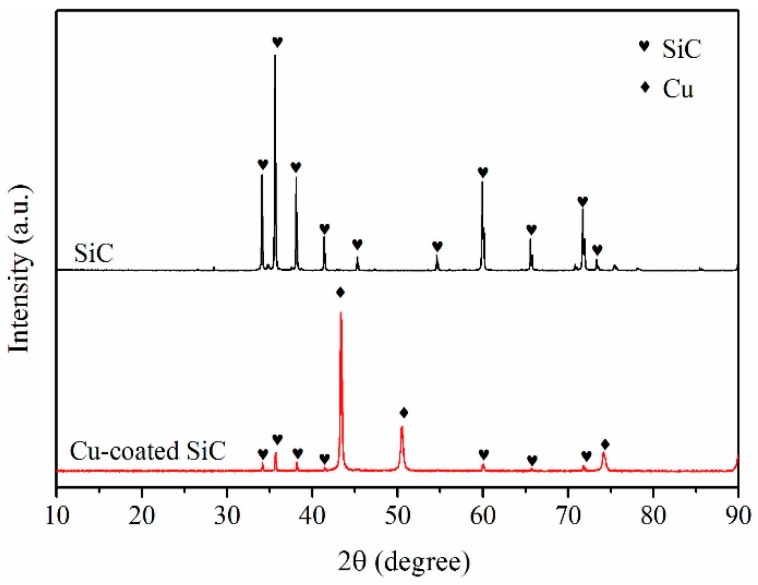
XRD spectra of the coated and uncoated SiC particles.

Figure 2 shows the XRD spectrum of the Cu-coated SiC particles, and compares it with the as-received SiC particles. It is clear that several sharp peaks corresponding to crystalline Cu appear on the spectrum of the SiC particles after electroless plating. This suggests that the nano-particles coated on the SiC particles shown in Figure 1e are copper particles/grains. Moreover, the Cu peaks are obviously higher than those for SiC, indicating that copper is the main phase on the surface of the particles, and that the SiC particles are well covered by the Cu coating.

### 3.2. Microstructure of the Fabricated BMGMC

**Figure 3:**
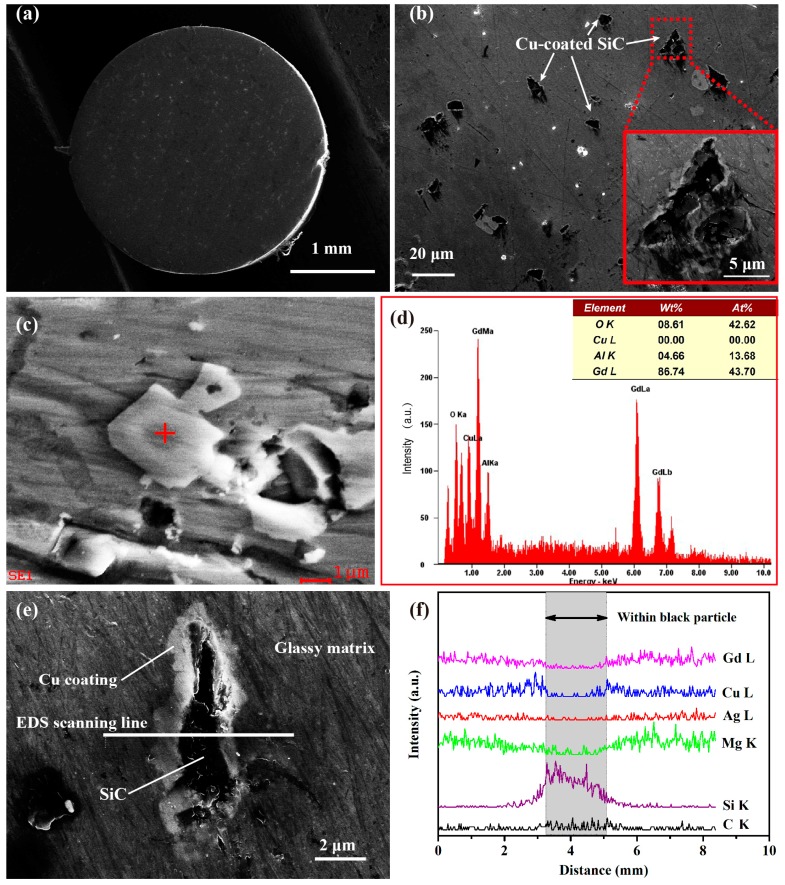
SEM images for typical as-cast BMGMC samples. (**a**) Overall view of the cross-section of an as-cast rod; (**b**) Low-magnification image of the sample with an inset showing the clustering of the SiC particles; (**c**) High-magnification image of the white particles; (**d**) EDS spectrum of the particle marked by red cross in (**c**); (**e**) High-magnification image of the sample showing the interface; and (**f**) EDS line-scanning spectra on the white line marked in (**e**).

Figure 3 shows typical SEM images of the cross-section of the as-cast BMGMC rod. From the overall view in Figure 3a, many particles can be observed, distributed evenly on the matrix. In the higher-magnification image shown in Figure 3b, the shape characteristics of the granular material, which are similar with that of SiC particles as shown in Figure 1, are clearly exposed, implying that the black phases are SiC particles. It is worth noting that most of these particles are well dispersed, except for a small number of them, which are assembled into a cluster, as shown in Figure 3b. In addition to the SiC particles, there are also some white particles in the cross-section of the sample, as shown in Figure 3a,c. The EDS spectrum performed on the particle marked by a red cross in Figure 3c is shown in Figure 3d. The contents of Gd, O and Al are obviously higher than for other elements, showing that the particle is probably gadolinium oxide, introduced through the use of aluminum foil in the preparation of BMGMC.

In addition, it also can be seen in Figure 3b,e that there is a thin white coating covering each SiC particle, including the particle in the cluster, as shown in the inset of Figure 3b. The thickness of the coatings was in the range of ~500 nm to 1 μm. This is consistent with the observed result of the electroless copper coating on the SiC particles. In order to confirm the phase of the observed thin coating, an EDS test upon the white line marked in Figure 3c was carried out, and the results are shown in Figure 3d. Within the black particle region, C and Si elements had increased, and the other elements had decreased significantly, indicating that the black particle is a SiC particle. For the white coating region, Cu content was obviously increased, while the other elements were not, demonstrating that the coating is mainly composed of Cu element. Meanwhile, the good dispersion of the SiC particles indicates that the wettability between Cu-coated SiC particles and Mg-based melt is good, which can be attributed to the presence of the Cu coating.

**Figure 4:**
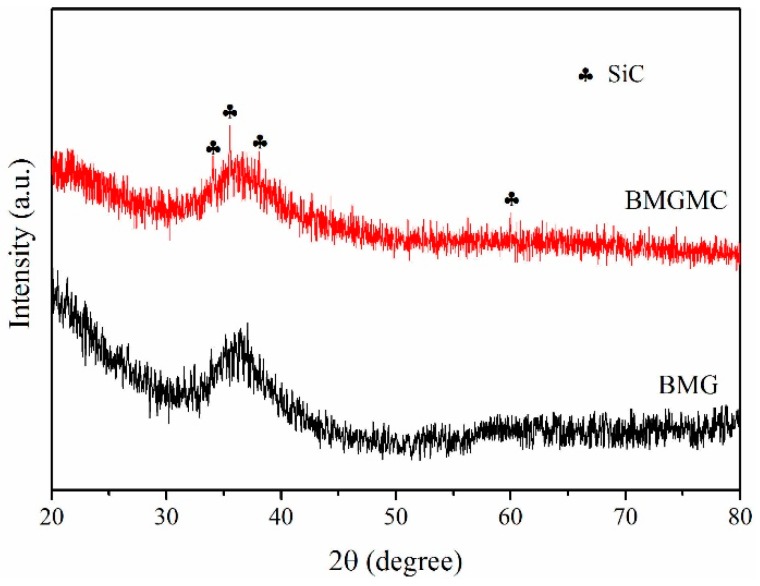
XRD spectrum of typical BMGMC sample compared with the BMG base alloy.

Figure 4 shows the XRD spectra of the BMGMC sample and the BMG sample, for further confirmation of the phase composition of the BMGMC. In comparison to the spectrum of Mg_54_Cu_26.5_Ag_8.5_Gd_11_ BMG, the spectrum of the BMGMC sample exhibits a difference, in that some small diffraction peaks corresponding to SiC appear. Although the diffraction peaks are not obvious, due to the small number of SiC particles, they can indicate the presence of SiC in the BMGMC sample. However, the diffraction phenomenon of Cu is not obvious, due to the fact that the amount of crystalline Cu contained in the BMGMC is too tiny to be detected by general XRD. In our opinion, the very thin electroless Cu coating is beneficial for obtaining a light-weight composite.

Furthermore, considering Figure 3 and Figure 4 in combination, there are no obvious crystallization phenomena to be found, indicating that the Cu coating and the SiC particles are not melted in the Mg_54_Cu_26.5_Ag_8.5_Gd_11_ liquid. In other words, the Cu, Si and C atoms have not diffused in the melt, changing the chemical composition of the glass-forming liquid. Therefore, the matrix composition of the composite is still within the optimum range in terms of glass-forming ability (GFA), enabling the formation of an all-amorphous matrix, and thereafter ensuring the high strength of the composite.

### 3.3. Mechanical Behavior of BMGMC

**Figure 5:**
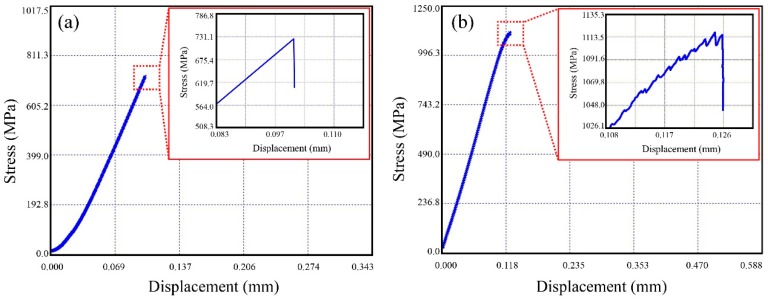
Typical engineering stress-displacement curves of (**a**) the BMG sample and (**b**) the BMGMC sample. The inset in each figure shows the enlarged view of the curve at the fracture point.

Figure 5 shows the compression test results of typical BMG sample and BMGMC sample. For the BMG sample, the stress-displacement curve showed no obvious yielding process, and the sample fractured at a stress of ~750 MPa, which is in agreement with the literature [29], and shows the obvious brittle deformation behavior in the BMG. For the BMGMC sample, the fracture strength was increased to over 1100 MPa with an increment of ~47%. This result is higher than that obtained for non-coated SiC particle-reinforced BMGMCs in a similar work [19]. Meanwhile, the fracture stage of the stress-displacement curve of the BMGMC sample is obviously different from that of the BMG sample. Before the fracture, the curve initially bends, and shows a serration flow feature as shown in the inset of Figure 5b. As is well known, serration flow is a universal phenomenon in ductile BMGs [30,31], but are rarely found in Mg-based BMGs and BMGMCs [32]. For example, although the addition of Mo induces great plastic strain in BMGMCs, no obvious serration behavior could be found in the stress-strain curves [33]. Therefore, the observed serration flow phenomenon indicates that the mechanical behavior of the BMGMC has been changed from having a typical brittle quality to a plastic quality.

**Figure 6:**
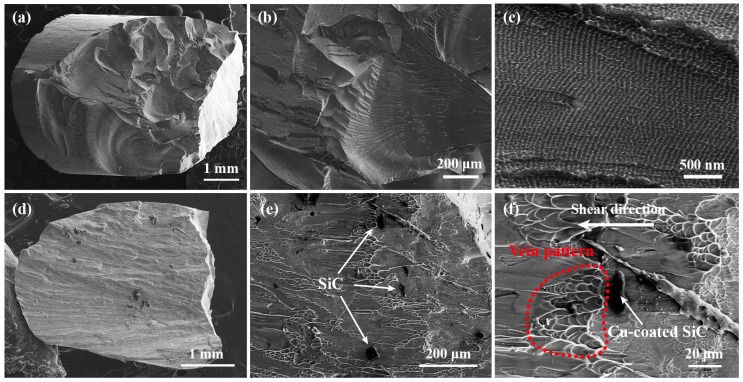
SEM images showing the typical fracture surface of (**a**–**c**) the BMG sample; and (**d**–**f**) the BMGMC sample.

Figure 6 displays the typical fracture morphologies of the BMG sample and BMGMC sample, respectively. In general, Mg-based BMG samples break into many fragments due to the brittle fracture [34]. Nevertheless, sometimes, big fragments can occasionally be found, and Figure 6a shows one in which the fracture surface has typical cleavage fracture features. In the higher-magnification images in Figure 6b,c, the whole fracture surface is composed of many smooth planes. On each smooth plane, many periodic corrugation morphologies in nano-scale can be observed as shown in Figure 6c. The size of the periodic corrugation in the present Mg-based BMGs is ~70 nm.

For the BMGMC sample, the compressed sample often fractures into two parts, and the fracture has unique shear features. Figure 6d presents an overall view of the fracture of the BMGMC sample. At macro scale, the fracture surface looks more flat, which is significantly different from the BMG sample. In the high-magnification image in Figure 6e, one can clearly see that there is no smooth cleavage plane or corrugated pattern. In contrast, an obvious vein-like pattern appears on the surface, together with a small number of SiC particles. The vein pattern is a typical fracture morphology that is often found in plastic BMGs [34,35,36,37], and is related to the formation of some viscous layers, as well as the shear band, during shear deformation [38,39]. The presence of the vein pattern indicates that the deformation of the composite sample changes from brittleness to toughness. In addition, the SiC particles are often found to be distributing after the vein pattern along the shear direction, as shown in Figure 6f. This indicates that SiC particles might stimulate the nucleation of the shear bands, leading to obvious plastic deformation behavior in the BMGMC sample.

## 4. Discussion

### 4.1. Role of Electroless Plating

For SiC particle-reinforced amorphous matrix composites, electroless copper plating plays an important role in modifying the morphology of SiC particle in order to reduce stress concentrations. Single SiC particles generally have a single crystalline structure; therefore, its strength and hardness are very high, making SiC an ideal wear-resistant material. However, the appearance of single-crystal SiC particles often exhibits very sharp edges and vertex angles, leading to greater stress concentration at the corresponding locations in the composite. Therefore, the appearance of SiC particles must be modified. In the present work, SiC particles were wrapped with a soft copper coating to passivate the sharp edges and corners of the particles, reducing the corresponding stress concentrations. It should be noted that the thickness of the copper coating was very small (<1 μm). Thus, the introduction of the Cu coating did not significantly increase the weight of the SiC particles. It is therefore conducive to the preparation of lightweight composite materials.

The electroless copper plating on SiC particles still faces many challenges. It has been found that smaller SiC particles (<10 μm) agglomerate together easily to form larger clusters during copper plating. This structure is not conducive to an optimal dispersion of SiC particles in the matrix, and is disadvantageous to increasing the dispersion-strengthening effect. Therefore, an electroless plating process suitable for fine SiC particles needs to be developed as soon as possible. In addition, the thickness of the electroless copper plating layer may be an important parameter for the performance control of the composite material. It still requires a lot of work to coat small SiC particles with a metal coating of different thicknesses and even in different chemical composition ranges.

### 4.2. Toughening Mechanism

The degree of brittleness in BMGs can be characterized by the characteristic plastic size, as well as the process-zone size at the tip of a sharp crack [40]. The process-zone size d can be evaluated by

(1) $$d=\frac{1}{6\pi }\cdot \frac{K_{c}^{2}}{\sigma_{y}^{2}}$$

where *K_c_* is the fracture toughness and *σ_y_* is the yielding strength, which can be replaced by fracture strength *σ_f_*. It has been shown that the Mg_59.5_Cu_22.9_Ag_6.6_Gd_11_ BMG has a fracture toughness *K_c_* of about 8.2 MPa·m^1/2^ [41]. Therefore, the process-zone size d of Mg_59.5_Cu_22.9_Ag_6.6_Gd_11_ BMG, which can be calculated using Equation (1), is about ~4 μm. This result suggests that the plastic deformation at the tip of a crack is highly limited; in other words, the accumulated elastic deformation energy cannot be sufficiently dissipated by plastic deformation. As a result, the typical plastic features, including the shear band and the vein-like pattern, cannot be found in Mg-based BMGs. Instead, brittle BMGs often show periodic corrugation at the nano-scale on the fracture surface, as shown, for example, in Figure 6c, which can be attributed to the Rayleigh wave that was formed during the fracture [40,42].

In accordance with the general strategy of material toughening, the plastic process zone at the tip of a crack should be enlarged to delay the rapid propagation of cracks. Given that d is an intrinsic property of the material, it cannot be directly increased without changing the chemical and microstructural composition. The strategy of coating SiC particles with a soft/plastic metal layer would be an effective method for increasing the process-zone size d in BMGMCs. In our opinion, the sharp corners of SiC particles easily become a potential sources of cracks in the MG matrix. The Cu coating around the SiC particles covers the corners, and can be regarded as a plastic zone ahead of the sharp crack (SiC particle), which is equivalent to improving the d value. Although this increment is very limited due to the small thickness of the thin coating (only about 1 μm), it is nearly 25% of the original value (~4 μm). In addition, the presence of the coating also reduces the angular sharpness of SiC particles and reduces the degree of stress concentration. Therefore, the rapid propagation of the crack is expected to be suppressed. The results shown in Figure 5 and Figure 6 also prove this point. Although the Cu-coated SiC particles did not significantly improve the plastic strain of the composite sample, the fracture strength was significantly improved. Moreover, the fracture process of the BMGMC sample obviously yielded characteristics such as serration behavior in stress-strain curves (see Figure 5) and vein-like patterning on the fracture surfaces (see Figure 6). This demonstrates that our strategy is feasible, and is expected to increase the shear-band formation and the plastic deformation abilities of BMGMC. However, the effect of electroless coating, which is performed on SiC particles, on the toughening of BMGMC still needs further research. It is necessary to confirm the best thickness for the electroless copper plating layer for improving the plasticity of BMGMC, which should combine the progress of studies on the electroless plating of SiC. Additionally, the best size for the Cu-coated SiC particles, along with the most suitable added amount to use, needs to be clarified.

## 5. Conclusions

Electroless copper plating is an effective method for increasing the interface wetting between SiC particles and the Mg-based metallic glass-forming melt, which is beneficial for the fabrication of SiC particle-reinforced Mg-based metallic glass matrix composites. The electroless copper coating is stable in the melt, and acts as a protective layer for SiC particles, preventing the interface reaction between SiC and the Mg_54_Cu_26.5_Ag_8.5_Gd_11_ melt. In addition, electroless plating can also modify the shape of SiC particles, improving the plastic process zone size at the tips of cracks, and decreasing the tendency to form stress concentration in the vicinity of the sharp edges and corners of the particles. As a result, the formation of shear cracks is suppressed by the Cu coating, instead by the nucleation of shear bands, leading to the increased plastic deformation ability of the BMGMC.
